# Editorial

**Published:** 1876-08

**Authors:** 


### Editorial.

### REPLANTING A TOOTH.

About the first of April last, Mr. M. called for examination of the left inferior second bicuspid. An abscess had formed about its root, which made a fistulous opening on the outside with the orifice about two thirds of an inch above the lower border of the jaw. This had been discharging daily for more than a year; this had been suffered to continue, because at no time had there been any appreciable pain and but little soreness of the tooth. Upon the surface there was considerable depression of the soft parts around the orifice, and some adhesion of this tissue to the jaw. This depression and adhesion seemed to be on the increase. This perhaps more than anything else induced the patient to seek relief. Advice had already been given to the effect that the tooth must be removed and the adhesion relieved by cutting. The tooth had upon its posterior surface a large cavity for such a tooth; this was indifferently filled with gold. The pulp had died some time after the tooth had been filled and after this the abscess formed.

After making an examination, and taking all the circumstances into account, we decided to remove the tooth, treat, fill and replace it.

This course was adopted, however, with considerable hesitancy from the fact that the systemic condition was not good, the vital forces not strong and these had not been preserved as they should have been.

There was no loss of gum or process, at least so far as the borders were concerned. The first bicuspid and first molar were both standing and in good condition.

The tooth in question was removed without difficulty. About one third of the root from the point was covered with the coagulated lymph material usually found in such cases. The treatment consisted in removing all this material and and scraping the root from the neck to the apex, depriving it of every vestige of soft tissue. The entire substance of the tooth presented a good color. The filling was removed, decay to some extent had taken place beneath it. The pulp chamber and canal were filled with decayed, offensive matter; all this was removed, the canal drilled entirely through, and the whole filled with gold. The operations upon and in the tooth occupied fifty-five minutes.

The clot of blood was now removed from the socket and the latter bathed with Extract of Hamamelis; the root was washed and moistened with the same preparation, and immediately replaced in the socket. Very little pain was experienced by the replacement, the teeth of the upper jaw were brought upon it till completely down to its proper place. There was a little soreness for two or three days, but no pain and within a week the soreness was all gone. Within two weeks mastication was performed upon this and the adjacent teeth as usual. The discharge from the fistula diminished in quantity from the time of the operation till it entirely ceased, which was about eight days. From that to the present time, over three months, there has not been an unfavorable symptom. The depression of the parts about the fistulous opening is relieved, the part is restored to its normal shape; a mere trace of adhesion remains which can only be detected by manipulating the parts.

This part of the work has been wholly accomplished by nature; no treatment whatever, except that already described. being employed. Several lessons may be learned from this case, one of which is the ability of nature to repair injuries when obstacles are taken out of the way. Another is the readiness, or avidity rather, with which nature clings to all that belongs to her, even to that which has hitherto been regarded as without life.

### THE OHIO STATE SOCIETY.

We would direct the special attention of those interested to the statements and suggestions of Dr. Porter on another page in this No. of the REGISTER, in reference to the present condition of our State Dental Society. His presentation of facts and figures are from the official documents; and his criticisms are just.

Now the Society would be more efficient, if it consisted of but twenty or even fifteen good active workers, than to have one or two hundred members, nine-tenths of whom are there, simply to have their names on the roll, and to make a little capital and prestige at home out of it. The man who upon being elected a member, returns home and has it announced by advertisement that he was elected a member of the State Society etc., etc. is unworthy of membership.

One of the absolute requirements for membership, should be, WORK, in and for the Society. It is no place for drones and they should not be tolerated.

The moment *any* man ceases to pay his dues without a reason satisfactory to the Society, his membership ought to cease and even those who pay their dues, and do nothing else, should hardly be admitted to membership; it might be well to have a kind of sub-membership for all such, especially if there seemed to be a prospect that they would become good active members, by and by.

Now let all the members think over this subject, and be prepared to take wise and judicious action.

### "A RAG FILE."

This is the name, and it is a good one too, that a lady patient gave to the strips of emery cloth used by a few operators for dressing and polishing gold fillings in the proximate surfaces of teeth. This little appliance is so good and efficient that it is deemed proper to call attention to it.

It is made of sheets the size of ordinary sand paper, with various grades of emery, The grades are marked on the sheets; the best suited for the dentist's use are No. 1, No. 0, and No. 00. The first, or No. 1, is quite coarse, and will cut down the surface of a filling more rapidly than a file, then follow this with the No. 0, or No. 00; the latter after being used a few moments gives a sufficiently fine finish for any ordinary case.

The strips should be cut from the sheets with a pair of strong shears and always parallel with the selvage. and should be from an eighth to a fourth of an inch wide. These strips are superior to any of the tapes that have been introduced, at least so far as we have observed.

---

### OBITUARY.

### DR. W. D. STONE.

It would be difficult to recall a case so sad and pitiable as that of Dr, W. D. Stone, whose death occurred in Louisville, Ky., on the 14th of June. He held almost national fame at one time; was noted throughout Kentucky. He will be remembered as a noble specimen of physical manhood. It was his boast, several years ago, that he was the only healthy dentist in Louisville. He was sixty-two years of age at the time of his death. Few practitioners ever enjoyed so remunerative practice as did Dr. Stone. He was apt, genial and pleasant in conversation. He was widely known, and above the average operators of his time. He adhered to those methods of practice which were acquired long ago and which seemed to him as giving best results. He was a man full of the world's knowledge, but not of letters. His handsome type of physical manhood was subjected to all the strains of bad habits and passions which bereft him of health, friends and relatives, and finally sent him to the asylum of the poor where he died, neglected, without a mourner, and without a tear falling over his grave. How bitter and how sad for poor Stone! He stood upon the Alpine heights of professional fame and dropped as an extinct rocket—a lesson that so many other bright masters of the profession may take heed. May we remember only his good deeds.

